# Supplementary material for: Very-high-frequency probes for atomic force microscopy with silicon optomechanics
Source: Microsyst Nanoeng. 2022 Mar 18;8:32. doi: 10.1038/s41378-022-00364-4 (PMC8931076; doi:10.1038/s41378-022-00364-4)
Supplement: Supplementary file 1 — Supplementary information [file 41378_2022_364_MOESM1_ESM.docx]

**SUPPLEMENTARY INFORMATION:**

**Very high frequency probes for atomic force microscopy with silicon optomechanics**

L. Schwab^1^, P. E. Allain^2^, N. Mauran^1^, X. Dollat^1^, L. Mazenq^1^, D. Lagrange^1^, M. Gély^3^, S. Hentz^3^, G. Jourdan^3^, I. Favero^2^ and B. Legrand^1,^^[[1]](#footnote-1)^

*^1^Laboratoire d’Analyse et d’Architecture des Systèmes, Université de Toulouse, CNRS UPR 8001, Toulouse, France*

*^2^Matériaux et Phénomènes Quantiques, Université de Paris, CNRS UMR 7162, Paris, France*

*^3^Université Grenoble Alpes, CEA, LETI, Minatec Campus, Grenoble, France*

**Fig. S1: Scanning electron microscopy images of a probe tip fabricated using the VLSI process on silicon.** (a) Top view of a 5-μm long probe tip. Red disk radius is 50 nm, green disk radius is 25 nm. Inset: tilted view of the probe tip. (b) Close view of the apex of the tip. Green disk radius is 25 nm. From the measurements the curvature radius of the apex of the tip is estimated to be smaller than 30 nm.

**Fig. S2: Optical transmittance spectrum of the waveguide coupled to the ring cavity.** (a) Broad band spectrum. The transmittance profile of the grating couplers produces a bell-shaped curve (red dashed line). Blue arrows indicate the optical modes of the ring cavity showing a free spectral range of 8.3 nm. (b) Optical mode at 1554.3 nm. A loaded quality factor of 10 500 and a contrast of 69% are deduced from the measurement. (c) Optical doublet at 1562.5 nm. When optical quality factors are higher that 10 000 typically, the optical mode appears split in a doublet caused by the degeneracy lifting of the clockwise and counter-clockwise modes propagating in the ring cavity. A loaded quality factor of 16 900 (resp. 12 000) and a contrast of 51% (resp. 47%) for peak 1 (resp. 2) are deduced from the measurement.

1. Author to whom correspondence should be addressed. Electronic mail: [bernard.legrand@laas.fr](mailto:bernard.legrand@laas.fr)
   T.: +33 5 6133 6811, F.: +33 5 6133 6300 [↑](#footnote-ref-1)
